# Mechanisms Involved in Pathological Succinate-Mediated Signaling

**DOI:** 10.3390/ijms27104328

**Published:** 2026-05-13

**Authors:** Bismarck Bernabe-Yepes, Cecilia Zazueta

**Affiliations:** Departamento de Biomedicina Cardiovascular, Instituto Nacional de Cardiología Ignacio Chávez, Juan Badiano No. 1 Tlalpan, Mexico City 14080, Mexico; bis_by@comunidad.unam.mx

**Keywords:** succinate, SUCNR1, DAMP, inflammation, biomarker

## Abstract

Succinate is a key intermediate of the Krebs cycle, which has increasing recognition for its roles beyond energy metabolism, including inflammation, cellular signaling, and metabolic regulation. Extracellular succinate, in particular, has been recognized as a signaling molecule that acts via the succinate receptor 1 (SUCNR1). SUCNR1-mediated signaling, however, demonstrates significant heterogeneity across various pathological contexts, resulting in varied and occasionally contradictory biological outcomes. This complexity underscores the context-dependent characteristics of succinate signaling and its significance in disease progression. This review will offer a comprehensive analysis of the signaling pathways activated during the interaction of succinate with its receptor in various tissues, as well as the potential of succinate regulation to ameliorate several pathological conditions.

## 1. Introduction

Inflammation is a tightly controlled process that reacts to different stimuli and releases mediators that activate cells and move inflammatory leukocytes to the site of damage, to remove or minimize harmful stimuli and eventually end acute inflammation [1]. Excessive local regulatory mechanisms may induce chronic systemic inflammation that impacts metabolism in remote tissues and organs, facilitating the emergence or progression of pathological conditions.

Regardless of the initial stimulus, the inflammatory response involves the following common mechanisms: (1) recognition of the stimuli by cell surface pattern receptors; (2) activation of inflammatory signaling pathways; (3) release of inflammatory mediators; and (4) recruitment of inflammatory cells to the site of injury [2].

Neutrophils and macrophages start the inflammatory response by using their pattern recognition receptors, namely Toll-like receptors (TLRs) and NOD-like receptors (NLRs). These receptors are activated by pathogen-associated molecular patterns (PAMPs) and damage-associated molecular patterns (DAMPs). Downstream inflammatory signaling cascades include the activation of IkB and MAP kinases, the modulation of transcription factors like NF-κB and AP-1 and the expression of pro-inflammatory cytokines and proteins [3].

DAMPs are endogenous danger signals released during cellular damage or stress, capable of activating the innate immune system. They consist of a diverse range of molecules, including nucleic acids, proteins, ions, glycans, and metabolites. Under normal physiological conditions, these molecules do not elicit an immune response. However, when cells are damaged or stressed, changes in their concentration, distribution, or in their physical and chemical properties turn them into immunogenic signals. Innate immune receptors then recognized immunogenic DAMPs, triggering inflammatory responses [4]. DAMP release is broadly classified into two main categories: passive release, typically resulting from cell death, and active release from viable cells, mainly through exocytosis. Most types of cell death are regulated processes, rather than purely passive events. Some cellular mechanisms that are involved in the release of DAMPs can still cause inflammation independently of actual cell death, indicating that cells can contribute to inflammation even while remaining viable [5].

## 2. Review Methodology

Databases Consulted: The main database consulted was PUBMED/Medline as a subject-specific database. Search Terms: Those included core concepts, subject headings and keywords, such as “mitochondrial DAMPS”, “succinate receptor signaling”, “succinylation in physiopatology” and “succinate metabolism in health and disease”. Time Span Covered: We include the most recent literature ranging between 5 and 10 years to guarantee relevance and currency. Some classical concepts described in the text, have earlier references. Inclusion and Exclusion Criteria: We included mainly experimental reports, but also small-case clinical reports [6] and large clinical studies [7] were incorporated. We did not include Editorials nor conference abstracts.

## 3. Mitochondrial Dysfunction in Inflammatory Damage

Mitochondria are unique double membrane-enclosed organelles that exert a wide variety of essential cellular functions. Beyond their well-established role in energy production via oxidative phosphorylation, mitochondria participate in many important homeostatic processes, such as growth factor signaling, differentiation, cell death, autophagy, hypoxic stress, regulation of intracellular calcium levels and innate immunity [8,9,10]. The link between inflammation and mitochondrial dysfunction is evidenced in the metabolic reprogramming that innate immune cells undergo in response to external stimuli. In stimulated macrophages and dendritic cells, glycolysis and the pentose pathway are upregulated, while oxidative phosphorylation is decreased [11]. This metabolic switch ensures quick ATP production and gives cytokine production the building blocks it needs [12]. Mitochondrial dysfunction has extensive repercussions on cellular pathophysiology due to its diverse roles, and it is increasingly acknowledged as a pivotal factor in various pathological conditions, including neurodegenerative diseases, metabolic syndromes, and cardiovascular disorders [10]. Moreover, experimental evidence supports a multifaceted relationship between inflammation and mitochondrial dysfunction as significant factors in these diseases [8,13,14]. DAMPs in mitochondria can activate different inflammatory signaling pathways [15]. Among them, succinate has garnered heightened interest due to its effects on the response of immune cells [16].

## 4. Succinate Metabolism in Mitochondria and ROS Production

Succinate is an intermediary metabolite of the tricarboxylic acid (TCA) cycle, produced from α-ketoglutarate, which after being decarboxylated to succinyl-CoA, serves as substrate for succinyl-CoA synthetase. Once produced, succinate is promptly converted to fumarate by succinate dehydrogenase (SDH) or Complex II, which is part of both the TCA cycle and the mitochondrial respiratory chain. In this reaction ubiquinone (UQ) is reduced to ubiquinol (QH_2_) and flavin adenine dinucleotide (FAD) to reduced flavin adenine dinucleotide (FADH_2_) [17].

In pathological states like cardiac ischemia, succinate accumulates within the mitochondria, primarily due to the reversal of SDH activity. During ischemia, when there is no enough oxygen, SDH works in reverse by using QH_2_ produced at complex I. This makes fumarate reduction to succinate more likely, and fumarate overflow from purine nucleotide breakdown and the reversal of the malate/aspartate shuttle keeps this going [18]. Consequently, the flow through the remaining components of the electron transport chain is greatly reduced. Due to impaired oxidative phosphorylation, ATP production drops, and ADP is turned into AMP instead, reflecting the cell’s energetic deficit.

When oxygen is available again after reperfusion, SDH rapidly oxidizes the succinate that has built up. But in the first seconds of reperfusion, especially the first minute, a delay in the regeneration of ADP from AMP occurs, which slows down the activity of ATP synthase. This impairs the flow of electrons through complex III, which causes further accumulation of QH_2_, being added to the coenzyme Q reduced by SDH but not efficiently consumed. Because ATP synthase is not using the proton motive force, the mitochondrial membrane potential becomes hyperpolarized and complex I operates in reverse. This is called reverse electron transport (RET) [18]. RET at complex I is a major source of superoxide, causing significant oxidative damage; when combined with calcium overload it promotes the opening of the mitochondrial permeability transition pore (MPTP), which leads to cell death associated with ischemia/reperfusion injury [19].

## 5. Extracellular Succinate

Under physiological conditions, succinate is found in the extracellular space of human plasma at concentrations usually range between 6.1 and 23.5 μM [20]. This baseline level shows its steady-state role in cellular metabolism, particularly within the TCA cycle. But in some cases of disease or stress, extracellular succinate levels can rise significantly [21].

As mentioned before, higher levels of extracellular succinate have been observed during tissue hypoxia or ischemia, when not enough oxygen is available to the mitochondria. A study conducted by Zhang and colleagues has provided compelling evidence that during the subsequent reperfusion phase, succinate is not only metabolized by the mitochondrial enzyme SDH, but it is also released into the extracellular environment. It has been demonstrated that as much as two-thirds of the succinate accumulated during ischemia can be exported from the cell into the extracellular space. These results highlight the dual fate of succinate during reperfusion and suggest that its release to the extracellular milieu may be important for signaling and tissue response after ischemia [22].

Succinate levels also rise during inflammation, because activated immune cells release it as part of their metabolic and signaling responses. Hypothermic conditions also can cause succinate to build up, probably because enzymes work more slowly and metabolic flux changes [3,22,23].

In metabolic disorders such as diabetes, where mitochondrial function is altered and anaplerotic flux is increased, extracellular succinate levels are also higher. Furthermore, diverse types of cancer and other disorders, exhibit increased succinate release attributable to metabolic reprogramming, including SDH dysfunction and increased dependence on reductive carboxylation [18,20,24,25].

**Figure 1:**
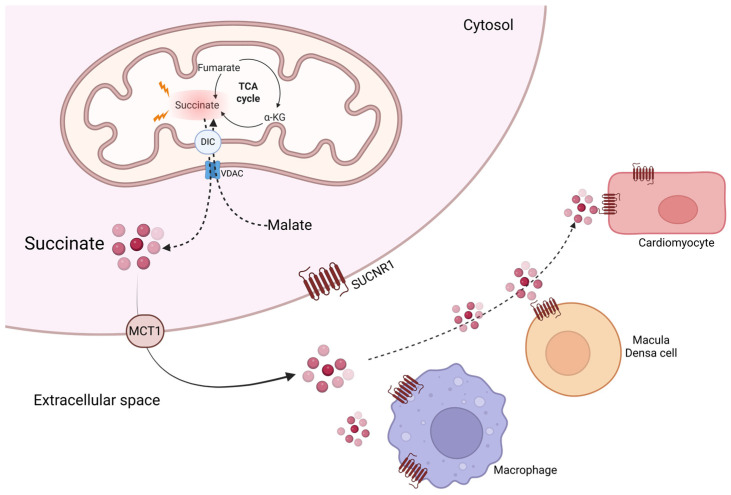
Accumulation of succinate and liberation to the extracellular space. Succinate accumulation is a metabolic hallmark of mitochondrial dysfunction. Under conditions such as hypoxia or impaired oxidative phosphorylation, succinate dehydrogenase can operate in reverse, promoting intracellular succinate buildup. Excess succinate is exported from the mitochondria via the dicarboxylate carrier in exchange for malate and subsequently released into the extracellular space through monocarboxylate transporters such as MCT-1. Extracellular succinate acts as a signaling metabolite by activating succinate receptor 1 (SUCNR1) in immune cells, cardiomyocytes, and renal cells. Succinate is a circulating metabolite; however, slight elevations in extracellular succinate can disrupt homeostasis and promote inflammatory responses. DIC: Dicarboxylate carrier, VDAC: Voltage-dependent anion channel, SUCNR1: Succinate receptor 1, MCT1: Monocarboxylate transporter 1. Created in BioRender. Chaverri, J. (2026). BioRender.com/4tdr13b (accessed on 8 April 2026).

The mitochondrial dicarboxylate carrier (DIC), located in the inner mitochondrial membrane exchanges succinate for malate, and in concert with the voltage-dependent anion channel (VDAC) releases this metabolite from the mitochondria to the cytosol [26], as shown in Figure 1.

At elevated cytosolic concentrations, succinate may function as a competitive inhibitor of prolyl hydroxylase domain enzymes (PHDs), which are responsible for hydroxylating hypoxia-inducible factor 1 alpha (HIF-1α) under normoxic conditions. Inhibition of PHD activity prevents HIF-1α degradation, resulting in its stabilization. This metabolic interference mimics the cellular response to low oxygen, a condition known as “pseudohypoxia”. Notably, HIF-1α enhances the production of IL-1β in macrophages [27,28].

Andrienko et al. [29] first suggested in 2017 that the proton-linked monocarboxylate carrier 1 (MCT1) may facilitate succinate export from the cell. This was based on a previous report that showed that the intracellular pH drops to <6.5 in the ischemic heart, converting more than 10% of succinate into its monocarboxylate form [30]. In addition, it has been previously demonstrated that AR-C155858, a potent and specific MCT1 inhibitor precludes the measurable proton-linked succinate transport at pH 6.0 in *Xenopus laevis* oocytes [31].

Based on this evidence, Prag and collaborators investigated whether the pH gradient of approximately one unit, established during reperfusion following ischemia, was related with succinate efflux mediated by MCT1 in an in vitro model. Their findings indicated that succinate release is reduced by approximately 90% upon the application of the specific MCT1 inhibitor (ARAR-C141990) during the reoxygenation of previously anoxic cardiomyocytes. Moreover, in an ex vivo mouse model, despite elevated succinate levels in cardiac tissue, the inhibitor exerted cardioprotective effects. They further validate the function of MCT1 in this process using a rat model with allelic insufficiency of the MCT1 gene. Succinate efflux from reperfused MCT1^+/−^ was effectively inhibited, providing strong evidence that MCT1 is the primary mediator of succinate export during reperfusion [32].

Nevertheless, another study indicated that the MCT1 inhibitor exacerbated tissue damage in an ex vivo model of ischemia/reperfusion, despite a reduction in succinate release into the effluent. It was suggested that succinate accumulated during ischemia within the mitochondria is oxidized by mitochondrial complex II (Cx-II), increasing ROS production, and promoting extensive cellular damage and cell death. Consequently, the concurrent inhibition of Cx-II activity significantly mitigated or even abolished these detrimental effects [33].

These observations underscore the complex role of succinate, suggesting that in addition to its intracellular accumulation as a metabolic intermediate under conditions of cellular stress, its release into the extracellular milieu promotes a shift toward receptor-mediated signaling. In this context, extracellular succinate activates SUCNR1, thereby broadening its impact on the modulation of various physiological and pathological processes. This change means that intracellular mechanisms, like metabolic rewiring and regulation driven by other mechanisms are more important during early metabolic imbalance. As succinate builds up and is exported, extracellular signaling becomes more important, connecting metabolic dysfunction to systemic inflammatory and adaptive responses.

## 6. Succinylation

High levels of succinate lead to the accumulation of succinyl-CoA, which causes global protein hypersuccinylation. This excessive succinylation frequently inhibits mitochondrial respiration-related enzymes, thereby intensifying metabolic dysfunction and the production of reactive oxygen species (ROS).

Succinylation is a newly identified post-translational modification (PTM) involving the covalent attachment of a succinyl group from succinyl-CoA to the epsilon-amino group of protein lysine residues. This modification is highly conserved across species and occurs in various cell compartments, although it is most prevalent within the mitochondria [34].

The process may be non-enzymatic, driven by high concentrations of succinyl-CoA and alkaline mitochondrial pH, or enzymatic, mediated by enzymes known as “writers” (succinyltransferases) such as KAT2A, CPT1A, and HAT1 [35,36,37]. The cellular effect is ultimately determined by the equilibrium between succinylation and desuccinylation processes. Desuccinylases, mainly Sirtuin 5 (SIRT5), in the cytosol and mitochondria, and Sirtuin 7 (SIRT7) in the nucleus, drive the reversible process [34,38].

Succinylation acts as a metabolic sensor that controls energy production and signaling in normal conditions. The heart, which has the highest concentration of succinyl-CoA, relies, relays on this PTM to control important processes like the TCA cycle, fatty acid metabolism, and oxidative phosphorylation [36,39].

Failing human cardiac myofibrils exhibit significant hyposuccinylation, a condition mainly attributed to a reduced mitochondrial succinyl-CoA pool. This decrease is caused by lower activity of α-ketoglutarate dehydrogenase (α-KGDH) and higher activity of succinyl-CoA synthetase, which accelerates succinyl-CoA turnover. As a result, less protein succinylation is linked to changes in sarcomeric mechanics, such as lower resting tension and a faster rate of activation in failing hearts. In contrast, the impairment of the desuccinylation machinery, as evidenced in SIRT5 knockout models, results in excessive protein succinylation, which facilitates hypertrophic cardiomyopathy and is associated with a deterioration in cardiac contractile function, indicated by reduced ejection fraction [34]. Pharmacological activation of SIRT5 might enhance outcomes in stroke and certain cardiovascular conditions by facilitating desuccinylation, reducing oxidative stress and restoring mitochondrial respiration [39].

## 7. SUCNR1 Signaling

SUCNR1, or GPR91, is a G protein-coupled receptor that is specific for succinate. Its identification represents an important step to understand metabolic signaling. SUCNR1 was first found in the proximal and distal renal tubules, where it is activated by physiological levels of succinate and triggers intracellular signaling through both Gi and Gq pathways [40]. At present, it has been detected in various organs, including the kidney, spleen, liver, heart, and small intestine [25].

SUCNR1 has a canonical structure consisting of seven transmembrane helices that span the cell membrane and an intracellular amphipathic helix. It also has a deep, hydrophilic, and electropositive pocket where ligands can bind. The receptor naturally recognizes succinate in a specific cis conformation [41].

Its activation by succinate is especially relevant under pathological conditions such as ischemia, hypoxia, and tissue damage, where succinate is released into the extracellular space, acting as a DAMP and triggering pro-inflammatory signaling pathways [42].

Two distinct succinate binding sites in SUCNR1 have been identified through unbiased molecular dynamic simulations and metadynamics simulations [43]. The occupancy of either of these sites function as a molecular switch that controls the receptor’s downstream signaling pathway. When succinate levels are low, only one binding site is typically occupied, keeping the receptor conformation in a stable conformation that preferentially activates Gq-proteins. This pathway is primarily associated with the mobilization of intracellular calcium. On the other hand, at high succinate levels, both sites are saturated, inducing a conformational shift that preferentially stabilizes Gi protein activation, thereby inhibiting adenylyl cyclase and reducing cAMP levels.

These findings have implications that go beyond basic biochemistry; they suggest that SUCNR1 acts as a metabolic “sentinel”. The receptor helps to maintain a balance between cellular energy production and protects against the excessive production of reactive oxygen species (ROS) and cell death during metabolic stress, by switching between Gq and Gi pathways when succinate levels rise. This mechanism offers a definitive explanation for the receptor’s dual role as pro-inflammatory in certain contexts and anti-inflammatory in others, contingent upon local pH and succinate concentrations [44].

## 8. Succinate–Succinate Receptor 1 Axis Effect in Pathological Conditions

The interaction between extracellular succinate and SUCNR1 is an area of active investigation [26], as this signaling axis appears to exert diverse and context-dependent effects on different organs and cell types. The succinate–SUCNR1 axis is an important regulator of cardiometabolic homeostasis, but its “double-edged” effects mean that a detailed molecular characterization is needed to keep it in mind as a possible drug target in clinical settings. In this context, it is pertinent to note that synthetic ligands are currently being investigated as potential drug targets for SUCNR1. In addition to metabolic agonists, such as malate, oxaloacetate, methylmalonate, itaconate and malonate, the synthetic non-metabolite agonists, SUC, cis-epoxysuccinate (CES) and (6-(4-(Trifluoromethoxy)phenyl)picolinoyl)-l-aspartic acid (compound **31**) have been identified as partial antagonists, whereas NF56-EJ40 is a SUCNR1-specific antagonist [43,45].

SUCNR1 agonists were discovered after the analysis of mini-libraries of nanomolar agonists for the receptor. These are backbone-modified analogs of succinate, which have an amide-linked hydrophobic moiety that is adjusted in a side pocket next to the succinate binding site [46]. Unlike succinate, these compounds are essential for elucidating the specific physiological role of SUCNR1, as they do not activate SDH, induce post-translational modifications or exert changes in cellular metabolism.

### 8.1. Heart

Chronic activation of SUCNR1 in ventricular cardiomyocytes is associated with the PLC–IP3–CaMKIIδ axis. This pathway induces the nuclear export of histone deacetylase 5 (HDAC5), which turns on hypertrophic genes [47,48]. In models of pressure overload, like pulmonary hypertension, succinate activates the PI3K/Akt pathway, increasing inflammation, myofibrillar reorganization, and significant fibrosis, all of which impair both contraction and diastolic function [49]. SUCNR1 activation triggers the MEK/ERK1/2 and CaMKIIδ/HDAC5 signaling cascades, inducing HDAC5 nuclear export and the re-expression of the fetal gene program of ANP, BNP, and Myh7, which promotes pathological hypertrophy. Moreover, SUCNR1 activation stimulates the renin–angiotensin system, exacerbating cardiac remodeling through systemic hemodynamic changes [48]. Extracellular succinate also promotes aberrant mitochondrial fission through two main pathways: the activation of PKCδ (which recruits Drp1 to the mitochondria) and ERK1/2 (which activates the mitochondrial fission factor, MFF) [50]. At the same time, PKCδ inactivates the pyruvate dehydrogenase (PDH) complex, blocking glucose oxidation and worsening the energy crisis during ischemic or hypoxia [51]. In this scenario, cAMP levels increased, leading to PKA activation, which modulates calcium transients by phosphorylating key handling proteins, specifically phospholamban (PLN) and the ryanodine receptor (RyR2). When exposed to millimolar concentrations of succinate, apoptosis is triggered by Caspase-3, ultimately exacerbating myocardial dysfunction and leading to the loss of myocytes [8].

In aging hearts, succinylation of the PKM2 enzyme at the Lysine 125 residue induces fibrosis. This modification promotes its dimerization and nuclear translocation, where it enhances HIF-1α activity and the expression of fibrogenic genes in fibroblasts [52].

When macrophages are under inflammatory or hypoxic stress, they accumulate succinate, which when released acts as an “inflammatory alarm,” binding to SUCNR1 on nearby effector cells. This binding stabilizes HIF-1α by inhibiting the PHDs that usually break it down. HIF-1α translocates to the nucleus and promotes the transcription of pro-IL-1β and NLRP3. HIF-1α positively regulates the NLRP3 inflammasome complex, which recruits and activates Caspase-1. This cleaves pro-IL-1β into IL-1β, increasing intravascular inflammatory responses and exacerbating plaque formation in atherosclerosis [53].

In models of heart failure with preserved ejection fraction, the succinate–SUCNR1–Gq axis activates AMPK. This enhances NAD^+^ biosynthesis and improves diastolic function, indicating that SUCNR1 functions as a sensor for energy deficits to reestablish homeostasis [54].

**Figure 2:**
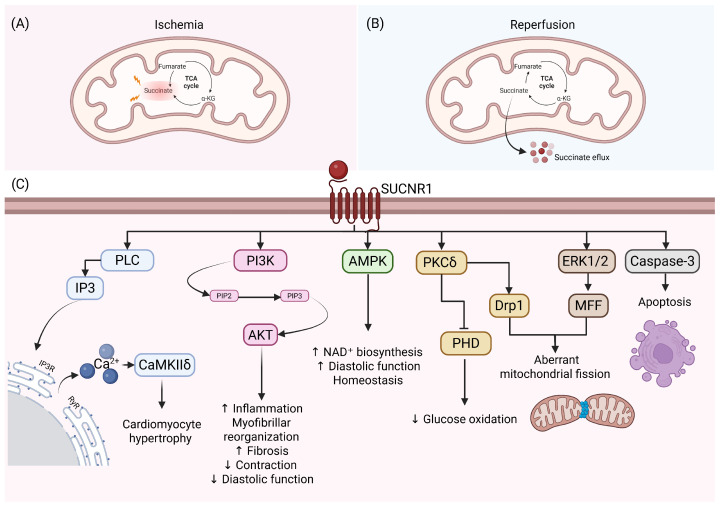
Succinate–SUCNR1 axis in the heart. (**A**) The primary mechanism underlying extracellular succinate generation in the heart occurs during ischemia. Under these conditions, mitochondrial dysfunction promotes reverse activity of succinate dehydrogenase, leading to the reduction of fumarate to succinate. This reverse electron transport results in a marked intracellular accumulation of succinate. (**B**) Upon reperfusion, succinate undergoes rapid oxidation as mitochondrial respiration is restored. However, despite its accelerated metabolism, a substantial fraction of accumulated succinate is released into the extracellular space through monocarboxylate transporters. (**C**) Once in the extracellular space, succinate can act as a signaling metabolite by interacting with neighboring cells. Specifically, it binds to succinate receptor 1 (SUCNR1) expressed on the surface of cardiomyocytes, triggering downstream signaling pathways that may contribute either to cardiac injury or to adaptive and protective responses, depending on the physiological context. SUCNR1: Succinate receptor 1, PLC: Phospholipase C, IP3: Inositol 1,4,5-trisphosphate, Ca^2+^: Calcium, CaMKIIδ: Calcium/Calmodulin-dependent Protein Kinase II delta, PI3K: Phosphoinositide 3-kinase, PIP2: Phosphatidylinositol 4,5-bisphosphate, PIP3: Phosphatidylinositol 3,4,5-trisphosphate, AKT: Protein Kinase B, AMPK: AMP-activated protein kinase, PKCδ: Protein Kinase C delta, PHD: Prolyl Hydroxylase Domain, Drp1: Dynamin-related protein 1, ERK 1/2: Extracellular Signal-Regulated Kinases 1 and 2, MFF: Mitochondrial Fission Factor. Created in BioRender. Chaverri, J. (2026). BioRender.com/gvxuvpc (accessed on 8 April 2026).

The contradictory nature of these findings incites a scientific discourse concerning the fundamental nature of SUCNR1. On one hand, its role in ischemia is clearly harmful. It acts as an “alarm signal” that accelerates reperfusion injury and inflammation by stabilizing HIF-1α and stimulating IL-1β production. Conversely, its capacity to activate AMPK in the context of chronic metabolic failure indicates that succinate may function as an adaptive therapeutic agent. Figure 2 shows that SUCNR1 has multiple signaling mechanism in heart tissue.

Controversial outcomes prompt inquiries regarding the conditions that determines whether the receptor associates with hypertrophy/reprogramming, the inhibition of lipolysis in adipose tissue, or apoptosis in cardiomyocytes. As previously stated, factors such as succinate concentration, where low levels may exert protective signaling and high levels may be harmful, along with exposure duration and the specific target cells (macrophages, fibroblasts, or cardiomyocytes), seem to be critical determinants of the ultimate biological response.

### 8.2. Kidney

Ischemic hypoxia and diabetic hyperglycemia are two main causes of succinate buildup outside cells. In diabetic hyperglycemia, excessive glucose levels overwhelm mitochondrial respiration. Metabolic disorders, including obesity and metabolic syndrome, alongside conditions such as oxidative stress and salt depletion, contribute to elevated succinate levels in renal tissue and urine, positioning it as a potential biomarker for kidney damage [55].

SUCNR1 is strategically localized in the apical membrane of Macula densa (MD), facilitating the measurement of succinate levels in the tubular fluid [56]. It is also present in glomerular endothelial cells, proximal and distal tubular epithelial cells, and immune cells, including macrophages [57]. This sensory positioning allows the receptor to translate metabolic signals into paracrine and systemic physiological responses [56].

In the juxtaglomerular apparatus, activation of the succinate–SUCNR1 axis triggers a signaling cascade that impacts renal hemodynamics and blood pressure regulation [58]. In MD cells, the pathway entails the activation of Mitogen-Activated Protein Kinases (MAPKs), particularly p38 and ERK1/2, resulting in the induction of cyclooxygenase-2 activity and the subsequent release of prostaglandin E2 [56].

Simultaneously, endothelial SUCNR1 activation increases intracellular calcium ([Ca^2+^]_i_) levels and enhances nitric oxide production. These mediators induce afferent arteriole vasodilation, contributing to glomerular hyperfiltration, and promote renin release from adjacent juxtaglomerular cells [56]. The final result is the renin–angiotensin system (RAS) overactivation, which drives systemic hypertension and early diabetic kidney pathology.

Within renal tubular epithelial cells, the axis mediates direct tissue injury via a pro-apoptotic signaling pathway. Succinate binding to SUCNR1 in these cells upregulates the ERK pathway, triggering the apoptotic mitochondrial pathway. This process increases the expression of pro-apoptotic markers, such as Bax and cleaved caspase-3, while lowering the levels of the anti-apoptotic protein Bcl-2. This results in significant structural damage, including the detachment of the brush border, tubular dilation, and cast formation, which are signs of progressive renal failure and chronic kidney disease [59].

Lastly, the succinate–SUCNR1 axis is fundamental in the progression of renal interstitial fibrosis because it changes the immune microenvironment. In macrophages, SUCNR1 activation initiates the p-Akt/p-GSK3β/β-catenin signaling cascade. This pathway promotes a shift from pro-inflammatory M1 macrophages toward an M2-like state of macrophages and causes the transcriptional upregulation of connective tissue growth factor. These profibrotic factors stimulate the proliferation and activation of renal fibroblasts via paracrine crosstalk, resulting in extensive extracellular matrix deposition. The axis is an important link between metabolic stress and long-term organ injury, such as tubulointerstitial fibrosis and the decline of the glomerular filtration rate [60].

### 8.3. Brain

The activation of SUCNR1 by succinate triggers a pro-inflammatory response by working synergistically with other pathways, such as TLR4, to enhance the expression of pro-inflammatory cytokines like TNF-α and IL-6. The mechanism behind this action involves the modulation of mitochondrial dynamics; specifically, SUCNR1 activation promotes mitochondrial fission (fragmentation), increasing ROS production and stimulating cellular motility and migration. This process has serious implications because it promotes the conversion of immune cells into a pro-inflammatory M1 phenotype, contributing to chronic inflammation and potentially leading to tissue damage or cell death, such as in neurodegenerative diseases [10].

SUCNR1 also mediates the pro-inflammatory response in the brain following ischemia–reperfusion injury. SUCNR1 activation synergistically triggers the NF-κB and NLRP3 signaling pathways, promoting Caspase-1 recruitment and the subsequent release of the cytokine IL-1β. This inflammatory cascade, which increases TNF-α and IL-17 levels, significantly exacerbates neuronal apoptosis. Consequently, suppressing SUCNR1 represents a potential therapeutic strategy to alleviate neuroinflammation [61].

### 8.4. Other Chronic Pathologies

The function of SUCNR1 was evaluated in conjunctival and corneal tissues, along with infiltrated inflammatory cells from patients with Mooren’s ulcer. The concentrations of IL-1β, MMP-13 and VEGF-A were increased in both tissues relative to healthy controls. In addition, IL-1β, MMP-13 and VEGF-A were detected in infiltrated inflammatory cells, suggesting a potential involvement of this receptor in the local immune response. Lipopolysaccharides (LPS) alone induced a robust inflammatory transcription of IL-1β mRNA, NLRP3, MMP-13 and VEGF-A. Co-stimulation with succinate substantially increased the expression of all these genes, indicating that SUCNR1 activation enhances the pro-inflammatory response. Together, these results demonstrate that succinate–SUCNR1 signaling acts as a potent amplifier of innate immune activation in this pathology [23].

In diabetic wounds, elevated glucose levels resulted in the accumulation of succinate and impaired the supportive interactions between M2 macrophages and epidermal stem cells. Enhanced SUCNR1 strengthened anti-inflammatory activity while also lowering intracellular succinate levels. Notably, SUCNR1 overexpression in macrophages maintained epidermal stem cells stemness and improved their migration through hepatocyte growth factor-dependent mechanisms. Inhibition of SDH further increased SUCNR1 expression, suggesting a compensatory upregulation of this receptor in response to altered succinate metabolism. At the signaling level, SUCNR1 activation triggered the pAkt/pGSK3β/β-catenin pathway, similarly than in renal fibrosis, highlighting the pivotal role of the SUCNR1 axis in regulating metabolic and inflammatory responses [62].

In ulcerative colitis (UC), chronic inflammation results in elevated levels of succinate, which actively participates in disease progression. Succinate affects the immune responses by enhancing Th17 cell activity and activating the NF-κB pathway through its specific surface receptor, SUCNR1. Engagement of this receptor also disrupts the glycolytic metabolism of intestinal epithelial cells, thereby exacerbating mucosal dysfunction and driving progression UC pathology [7].

In mice with necrotizing enterocolitis (NEC), a disease in which intestinal injury led to high mortality rate, elevated succinate levels disrupt the balance between anti-inflammatory and pro-inflammatory mediators. It was observed that SUCNR1 expression changes according to succinate concentration. Oral succinate administration also upregulates HIF-1α expression in a dose-dependent manner. Increasing succinate levels enhance the protein expression of both SUCNR1 and HIF-1α, supporting the notion that succinate contributes to NEC pathogenesis [6].

In rheumatoid arthritis, succinate has also been identified as a crucial immunometabolite that links mitochondrial dysfunction to chronic inflammation. When exposed to LPS or local hypoxia, macrophages shift from oxidative phosphorylation to aerobic glycolysis, leading to the intracellular accumulation of succinate. After it builds up, succinate is exported from the mitochondria to the cytosol and eventually to the extracellular space. Intracellularly, elevated succinate induces a state of “pseudohypoxia” by inhibiting PHDs, stabilizing HIF-1α. This stabilization triggers the transcription of potent pro-inflammatory genes, most notably IL-1β and VEGF [63]. Outside the cells, succinate binds to its receptor SUCNR1, which is found on the surface of immune cells, synovial fibroblasts, and endothelial cells, amplifying the inflammatory response in a paracrine and autocrine manner [64].

The downstream effects of this signaling cause the structural damage seen in rheumatoid arthritis. The stabilized HIF-1α and activated SUCNR1 synergistically promote synovial angiogenesis. The extra blood vessels in the inflamed tissue provide more oxygen and nutrients, but also facilitates leukocyte recruitment and pannus formation, which eventually destroys adjacent cartilage and bone. Succinate–SUCNR1 signaling in dendritic cells orchestrates their migration to lymph nodes, where they drive the expansion of Th17 cells. These cells are critical mediators of bone erosion, infiltration of neutrophils, and mechanical hyperalgesia [6,7].

In addition to systemic inflammation, the succinate pathway directly causes local cartilage destruction by chondrocyte pyroptosis. In the acidic rheumatoid arthritis microenvironment, acid-sensitive ion channel 1a (ASIC1a) is activated, triggering calcium influx. This influx, mediated by the Ca^2+^/CaMKK2/AMPK pathway, increases SDH activity and the production of ROS, leading to gasdermin D-mediated pyroptosis and irreversible cartilage degradation [65].

Research indicates that the activation of SUCNR1 can initiate distinct downstream pathways—including pro-inflammatory, metabolic, and fibrotic responses—depending on the tissue microenvironment and the pathological stimulus involved, as shown in Table 1. Although evidence is accumulating, the comprehensive understanding of SUCNR1-mediated signaling and its physiological and pathological significance remains insufficient, underscoring the necessity for further mechanistic characterization.

## 9. Extracellular Succinate Levels as a Potential Biomarker

In a prognostic study conducted by Angelo and colleagues, the findings indicate that plasma succinate is at least as effective as plasma lactate in predicting mortality among critically ill patients, demonstrating exceptional sensitivity and specificity. These findings highlight succinate’s potential clinical utility as a prognostic indicator in critical care settings, like trauma and hemorrhage-related death [66].

Conversely, circulating succinate has emerged as a significant metabolic marker in patients with type 2 diabetes undergoing bariatric surgery. Lower baseline succinate levels were strongly associated with higher remission rates at both 1 and 2 years post-surgery. Succinate alone showed predictive power comparable to traditional clinical scores, underscoring its relevance as a key indicator in metabolic recovery [67]. Succinate is a relevant biomarker of the severity of ischemia–reperfusion injury, and its release into the coronary effluent during early reperfusion is a specific indicator of mitochondrial membrane damage. Furthermore, succinate levels measured during early reperfusion demonstrate a strong negative correlation with subsequent cardiac functional recovery, offering superior specificity for predicting graft viability compared to conventional markers of myocardial cell death [68].

Succinate serves as a metabolic biomarker in organ transplantation, reflecting the extent of ischemic insult and predicts subsequent graft dysfunction across mouse, pig and human. During periods of warm and cold ischemia, succinate accumulates significantly due to the metabolic shifts that occur when oxygen is depleted, reaching plateau levels within 30 min of warm ischemia. Quantifying succinate levels is relevant for predicting post-transplant outcomes, as its concentration correlates directly with the severity of myocardial injury and the extracellular release of DAMPs, such as mitochondrial DNA and troponin. Because succinate levels are sensitive to the inevitable periods of warm ischemia during organ retrieval and anastomosis, monitoring its accumulation provides a real-time assessment of graft viability and the cumulative metabolic burden experienced by the organ. Furthermore, the ability to ameliorate IR injury by inhibiting succinate accumulation or oxidation—for instance, through the administration of malonate—underscores the importance of succinate as both a diagnostic biomarker and a therapeutic target for improving transplantation success [69].

Extracellular succinate also serves as a potent paracrine and autocrine driver of cancer metastasis. Succinate modulates the tumor microenvironment by inducing angiogenesis via ERK1/2/STAT3 activation and polarizing macrophages into tumor-associated macrophages (TAMs), both of which facilitate cancer dissemination, representing a critical pathological factor and a potential theranostic biomarker, as its elevated serum levels correlate with increased metastatic risk and poor prognosis [24]. In succinate dehydrogenase subunit B-mutated pheochromocytomas and paragangliomas, serum succinate serves as a robust oncometabolic biomarker, allowing for the identification of tumor-free asymptomatic carriers, offering a faster and more accessible diagnostic tool than next-generation sequencing, in addition of metastatic disease extension [70].

Blood samples can be used to measure extracellular succinate, which is a very sensitive indicator due to it rises quickly during acute ischemic events. Additionally, as a metabolite that persists elevated in chronic conditions, its concentrations have been associated with the extent of tissue damage across various pathologies, with higher extracellular concentrations correlating with greater injury. Quantitative mass spectrometry-based metabolomics can reliably detect extracellular succinate, providing rapid and robust results [66]. Nonetheless, more thorough and comprehensive research is required to ascertain its diagnostic superiority relative to current biomarkers, particularly through direct comparisons of sensitivity and specificity. Extracellular succinate is a promising candidate biomarker; however, further validation is necessary to establish its clinical relevance in future diagnostic applications.

## 10. Conclusions

Extracellular succinate is linked to various metabolic processes and is acknowledged as a principal regulator of inflammatory responses, alongside energy and metabolic homeostasis. The interaction between succinate and its G-protein-coupled receptor, SUCNR1, represents a complex paradigm in cardiovascular biology. In this context, succinate goes beyond being just a Krebs cycle intermediate and becomes act as a metabolic hormone or signaling molecule. Depending on the pathophysiological environment—such as ischemia, pressure-induced hypertrophy, or chronic heart failure—activation triggers intracellular cascades that can lead to either protective metabolic adaptation or cell death and fibrosis.

SUCNR1 activates diverse intracellular signaling pathways capable of triggering both pro-inflammatory and anti-inflammatory responses. This functional diversity may depend on the concentration duality of succinate signaling. SUCNR1’s capacity to detect and react to variations in extracellular succinate establishes a mechanistic framework that correlates metabolic status with context-dependent cellular outcomes, ultimately elucidating its dual role in inflammation, tissue remodeling, and disease progression.

These effects influence a wide spectrum of pathophysiological conditions, establishing this pathway as a focal point for research in multiple diseases. This interest stems primarily from the involvement of the same receptor in different mechanisms of injury across diverse tissues, underscoring the necessity for a more profound comprehension of the interaction between extracellular succinate and SUCNR1, as well as the need to further clarify these effects in a context-dependent manner, tailored to the specific physiological or pathological environment.

## Figures and Tables

**Table 1:** Tissue-specific signaling driven by the succinate–SUCNR1 axis.

Tissue	Model	Pathological Condition	Signaling	Outcome
Heart	In Vitro	Succinate administration (1 mmol/L)	MEK/ERK1/2 CaMKIIδ/HDAC5	Cardiomyocyte hypertrophy [48]
	In Vivo	Pulmonary hypertension	PI3K/Akt	Inflammation, myofibrillar reorganization and fibrosis [49]
	In Vivo/ In Vitro	Aging heart	PKM2 succinylation	HIF-1α activity and the expression of fibrogenic genes [52]
	In Vivo/ In Vitro	Ischemic injury	PKCδ and ERK1/2	Aberrant mitochondria fission [50]
	In Vitro	Succinate administration (10 mmol/L)	PKA	Caspasa-3 activation leading Apoptosis [8]
	In Vivo/ In Vitro	Heart failure	AMPK	Increase NAD biosynthesis and diastolic function [54]
Kidney	In Vivo	Diabetes mellitus (JGA)	Increase intracellular Ca^2+^, nitric oxide and PGE2	Increase blood pressure [58]
	In Vivo/ In Vitro	Diabetes mellitus (MD)	MAPKs/ERK1/2/ COX-2	Increase blood pressure [56]
	In Vivo/ In Vitro	Succinate administration (500 μM)	ERK/BAX/Caspasa-3	Apoptosis, detachment of the brush border, tubular dilation and cast formation [59]
	In Vivo/ In Vitro	Succinate administration (500 μM)	p-Akt/p-GSK3β/β-catenin	Fibrosis and decrease glomerular filtration rate [60]
Brain	In Vivo/ In Vitro	Succinate administration (5 mM)	TLR4/DRP1	Mitochondrial fission and increasing ROS production [10]
	In Vitro	Hypoxic-glucose deprivation	NF-κB/NLRP3	TNF-α, IL-1β e IL-17 increased pro-inflammatory response [61]

An overview of the signaling pathways activated in the heart, kidney, and brain under distinct pathological conditions. Depending on the context of injury, these pathways can trigger specific molecular and cellular responses that differentially contribute to tissue dysfunction and damage. The variability in signaling reflects the complexity of organ-specific responses, highlighting how similar stimuli may lead to distinct pathological outcomes across tissues. MEK: Mitogen-Activated Protein Kinase Kinase, ERK1/2: Extracellular Signal-Regulated Kinases 1 and 2, CaMKIIδ: Calcium/Calmodulin-dependent Protein Kinase II delta, HDAC5: Histone deacetylase 5, PI3K: Phosphoinositide 3-kinase, Akt: Protein Kinase B, PKM2: Pyruvate Kinase M2, HIF-1α: Hypoxia-inducible factor 1 alpha, PKCδ: Protein Kinase C Delta, PKA: Protein Kinase A, AMPK: AMP-activated protein kinase, PGE2: Prostaglandin E2, JGA: Juxtaglomerular Apparatus, MD: Macula Densa, MAPKs: Mitogen-Activated Protein Kinases, COX-2: Cyclooxygenase-2, BAX: BCL2-Associated X Protein, p-GSK3β: Phosphorylated Glycogen Synthase Kinase 3 Beta, β-catenin: Beta-Catenin, TLR4: Toll-Like Receptor 4, DRP1: Dynamin-Related Protein 1, NF-κB: Nuclear Factor Kappa B, NLRP3: NLR Family Pyrin Domain Containing 3, TNF-α: Tumor Necrosis Factor Alpha, IL-1β: Interleukin 1 Beta, IL-17: Interleukin 17.

## Data Availability

No new data were created or analyzed in this study. Data sharing is not applicable to this article.

## Abbreviations

The following abbreviations are used in this manuscript:

TLRs	Toll-like receptors
NLRs	NOD-like receptors
PAMPs	Pathogen-associated molecular patterns
DAMPs	Damage-associated molecular patterns
NF-κB	Nuclear factor kappa-light-chain-enhancer of activated B cells
AP-1	Activator Protein-1
ATP	Adenosine triphosphate
TCA	Tricarboxylic acid
SDH	Succinate dehydrogenase
UQ	Ubiquinone
QH_2_	Ubiquinol
FAD	Flavin adenine dinucleotide
FADH_2_	Reduced flavin adenine dinucleotide
ADP	Adenosine diphosphate
AMP	Adenosine monophosphate
RET	Reverse electron transport
MPTP	Mitochondrial permeability transition pore
DIC	Mitochondrial dicarboxylate carrier
VDAC	Voltage-dependent anion channel
SUCNR1	Succinate receptor 1
PHDs	Prolyl hydroxylase domain enzymes
HIF-1α	Hypoxia-inducible factor 1 alpha
MCT1	Monocarboxylate transporter 1
Cx-II	Mitochondrial complex II
ROS	Reactive oxygen species
PTM	Post-translational modification
SIRT5	Sirtuin 5
SIRT7	Sirtuin 7
α-KGDH	α-ketoglutarate dehydrogenase
HDAC5	Histone deacetylase 5
PDH	Pyruvate dehydrogenase
PLN	Phospholamban
RyR2	Ryanodine receptor
MD	Macula densa
UC	Ulcerative colitis
NEC	Necrotizing enterocolitis
